# Policy Development for Environmental Licensing and Biodiversity Offsets in Latin America

**DOI:** 10.1371/journal.pone.0107144

**Published:** 2014-09-05

**Authors:** Ana Villarroya, Ana Cristina Barros, Joseph Kiesecker

**Affiliations:** 1 The Nature Conservancy, Boulder, Colorado, United States of America; 2 The Nature Conservancy, Brasilia, Brazil; 3 The Nature Conservancy, Fort Collins, Colorado, United States of America; Instituto de Pesquisas Ecológicas, Brazil

## Abstract

Attempts to meet biodiversity goals through application of the mitigation hierarchy have gained wide traction globally with increased development of public policy, lending standards, and corporate practices. With interest in biodiversity offsets increasing in Latin America, we seek to strengthen the basis for policy development through a review of major environmental licensing policy frameworks in Argentina, Brazil, Chile, Colombia, Mexico, Peru and Venezuela. Here we focused our review on an examination of national level policies to evaluate to which degree current provisions promote positive environmental outcomes. All the surveyed countries have national-level Environmental Impact Assessment laws or regulations that cover the habitats present in their territories. Although most countries enable the use of offsets only Brazil, Colombia, Mexico and Peru explicitly require their implementation. Our review has shown that while advancing quite detailed offset policies, most countries do not seem to have strong requirements regarding impact avoidance. Despite this deficiency most countries have a strong foundation from which to develop policy for biodiversity offsets, but several issues require further guidance, including how best to: (1) ensure conformance with the mitigation hierarchy; (2) identify the most environmentally preferable offsets within a landscape context; (3) determine appropriate mitigation replacement ratios; and (4) ensure appropriate time and effort is given to monitor offset performance.

## Introduction

Over the next two decades, governments and companies will invest unprecedented sums – well over 20 trillion dollars – in development projects around the world, from Argentina to Zambia. Rapidly developing countries are making trillion dollar investments in infrastructure. For example, Latin America is in the midst of unprecedented and sustained growth in development as worldwide demand for the region's mineral, agricultural, and energy wealth grows [1], [2]. The region will need to construct more roads, energy facilities, and mines as this economic development continues. To be sustainable it is important to ascertain how this development can be done in a way that minimizes impacts and maximizes the benefits to nature and people. It will require that we find ways to balance the seemingly conflicting goals of improving infrastructure, increasing food production, and expanding access to reliable energy and housing while also preserving and protecting the biodiversity and ecosystem services of the region. To simultaneously achieve these goals will be challenging and require that development is complemented by public and private investments to prevent the loss of biodiversity and ecosystem services.

Environmental licensing processes, such as Environmental Impact Assessment (EIA) play a critical role in controlling the way development projects result in damage to the environment. In most countries developers are required to get an environmental license before development activities can be implemented, and currently EIA has been legally adopted in almost all countries in the world [3]. Obtaining such permit usually depends on the way the predicted negative impacts will be mitigated, or depends on the fulfillment of additional requirements set by the licensing authority. EIA is a systematic, iterative process that examines the environmental consequences of planned developments and emphasizes prediction and prevention of environmental damage [4]. The mitigation of environmental impacts is thus a key stage of the environmental impact assessment process and lies at its core [5]. Practitioners seek to reduce impacts through application of the mitigation hierarchy: avoid, minimize, restore, and offset [6]. To avoid impacts on biodiversity, measures are taken to prevent creating impacts from the outset, such as careful spatial or temporal placement of elements of infrastructure. In minimization, measures are taken to reduce the duration, intensity, and/or extent of impacts that cannot be completely avoided. In restoration, measures are taken to rehabilitate degraded ecosystems or restore cleared ecosystems after impacts that cannot be completely avoided and/or minimized. To offset impacts measures are taken to compensate for any residual adverse impacts that cannot be avoided, minimized, and/or restored. Offsets can take the form of positive management interventions such as restoration of degraded habitat, arrested degradation or averted risk, or protecting areas where there is imminent or projected loss of biodiversity [7], [8]. Attempts to meet biodiversity goals through application of the mitigation hierarchy have gained wide traction globally with increased development of public policy, lending standards, and corporate policy. In the public policy sector there are approximately 45 compensatory mitigation programs for biodiversity impacts worldwide, with another 27 programs in development [9]. In the financial sector, major institutions including the International Finance Corporation (IFC) and more than 70 Equator Principles financial institutions that base their requirements on IFC's Performance Standards are requiring projects they finance to adhere to the mitigation hierarchy. This means they should seek to avoid impacts on biodiversity and ecosystem services or - where this is not possible - to minimize or restore them. In critical habitats, this also means achieving net gains of biodiversity values for which these habitats have been designated. European Bank for Reconstruction and Development (EBRD) also has similar requirements [10]–[12]. As new performance standards and public policies drive mitigation biodiversity goals from a voluntary objective into the sphere of compliance, businesses (especially mining companies) are increasingly adopting it into corporate biodiversity management policies and mitigation practices as a normal way/cost of doing business [13]–[15].

**Figure 1:**
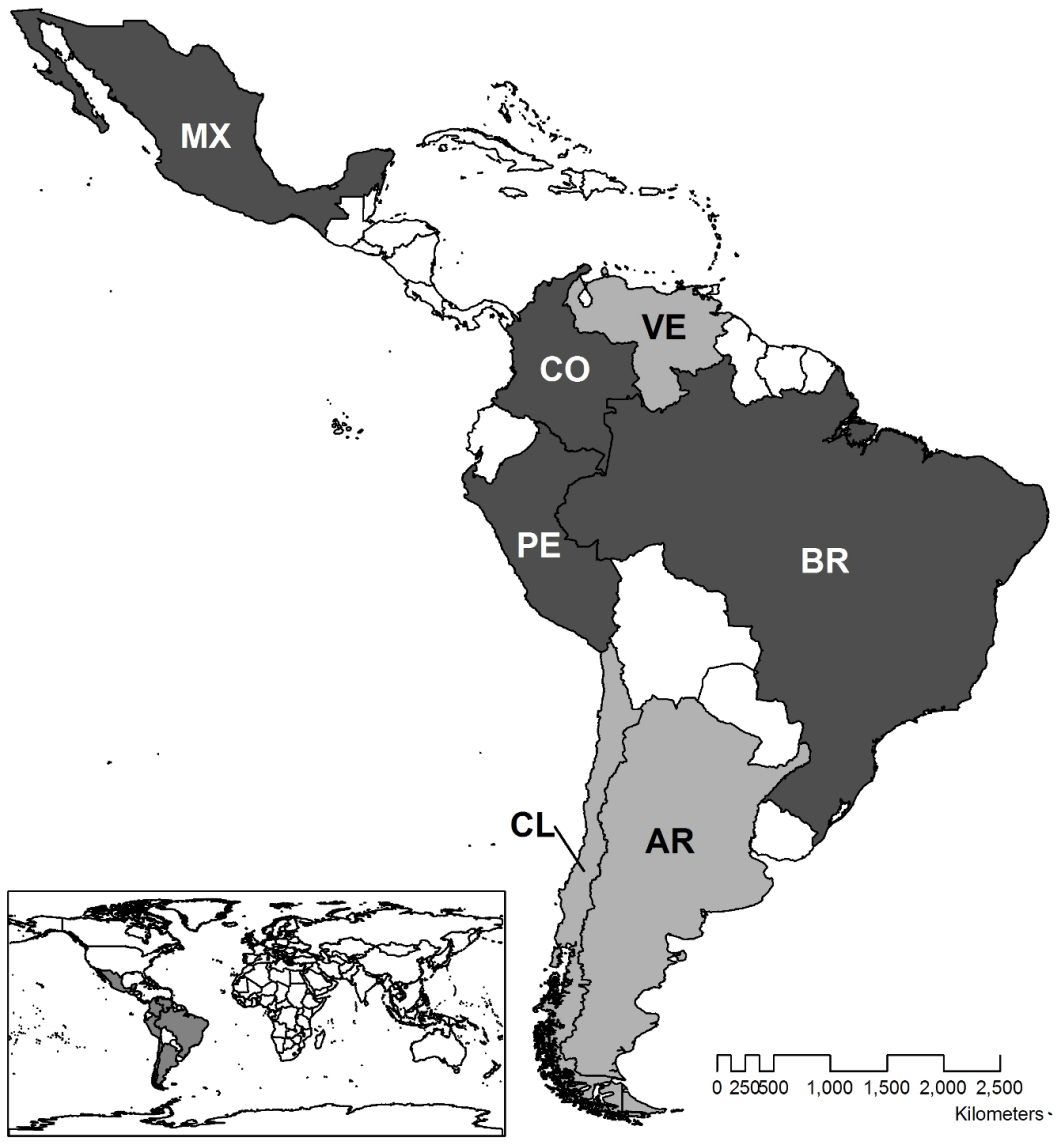
Countries selected for the study (in color). In dark grey, countries for which offset frameworks have been established. Countries' names have been abbreviated to the codes set by ISO 3166.

With interest in biodiversity offsets increasing in Latin America, we seek to strengthen the basis for policy development through a review of major environmental licensing policy frameworks in Argentina, Brazil, Chile, Colombia, Mexico, Peru and Venezuela (Figure 1). We focused on these countries because they represent ∼85% of the area of all Central and South America and ∼80% of the population of the region. Since we relied mainly on colleagues to identify and interpret policy documents we focused on countries where The Nature Conservancy has country level programs and staff available. We recognize the limitation of this approach but also do not consider this sample of countries to be a random sample intended to capture broader patterns in other countries found in the region. By comparing the goals, approaches, and key issues highlighted in these frameworks, and distilling important commonalities and differences, our aim is to provide guidance to countries that have not yet developed frameworks and to support improvements in existing policies. The frameworks selected for review include both established offset programs and rapidly emerging policies. With this analysis we sought to explore and analyze the role mitigation hierarchy and, more specifically, offsets are given in different Latin-American legal frameworks. First we conducted a broad review of policies related to the environmental licensing process, because the ecological effectiveness of mitigation depends heavily on the existence of strong environmental laws and regulations [16]. We then reviewed current offset frameworks from the selected countries. Finally we highlight negative and positive aspects of each countries mitigation frameworks as a guide to improve existing tools or proposal of new ones.

## Methods

We focused our review on national (federal) level policies to assess how current requirements would affect implementation of the mitigation hierarchy and promote positive environmental outcomes. State and provincial policies have not been included in this study. Although they are necessary to respond to local environmental contexts, the paper focuses on national policies because (a) the constitution of Chile, Colombia, Peru only allow for all laws to be established at the national level [17]–[19]. In addition in Venezuela environmental laws are only made at the national level [20]. Decisions on environmental licensing in Mexico are context dependent with large scale impacts (e.g. oil and gas, large hydropower, forest clearing, roads and railways) regulated at the federal level while localized environment impacts (e.g. urban expansion, small hydropower) are made at the state level [21], [22]. (b) National/federal policies often establish a common base for more specific documents such as state or provincial policies [23], [24]. (c) Infrastructure projects are often large and may affect more than one province or state. Thus we decided to focus on national level policies that would govern these types of projects. We sought to include three primary sources to gather and assess existing policies: 1. Official websites of each country's Ministry of the Environment (or equivalent agency), and any official agencies involved with the country's environmental licensing processes. 2. Published articles and reports about EIA and offset procedures in the selected countries. 3. Interviews with persons directly engaged in the mitigation agenda in each country. These interviews also helped ensure we interpreted the legal texts correctly. The interviews also helped confirm that all relevant legal texts had been selected and that we were not missing any information. For a complete list of sources used in our analysis see Table S1 and Appendix S1. We focused our analysis on existing policies and laws but we also included the new offsets law in Peru, that is about to be signed into law.

While a policy analysis may provide interesting and relevant information, it also has limitations that cannot be overlooked [25]. We acknowledge that environmental policies are numerous, varied and constantly changing, and the information they contain can be sometimes misinterpreted. Thus, even though all effort has been made to find and comprehensively review all relevant policies, we acknowledge that there is a chance that some regulations or information were missed. In those cases, we state that “no information has been found” instead of “no information exists on the subject”. Also, we must keep in mind that policies are in most aspects qualitative and difficult to compare and/or evaluate in a standard way that leaves little place to subjective interpretation. We have tried to overcome this handicap as much as possible by setting a list of specific and well defined questions to answer when reviewing the selected texts (see below).

### Review of Policies Related to Application of the Mitigation Hierarchy

To assess how a countries environmental licensing process would promote positive conservation outcomes we have reviewed national legal texts related to EIA processes and mitigation for infrastructure projects. We focused on general environmental policies, such as environmental acts as these laws often make provisions for EIA, or establish how mitigation activities are carried out. We also paid attention to sector-specific policies, since it is common to find specifications on how EIA shall be carried out for certain types of development (i.e. mining) projects, or require specific mitigation measures for particular types of development. In addition we also examined habitat/area-specific policies (e.g. wetlands), since sometimes they include provisions related to impact mitigation [26].

Although our analysis examined some aspects of the EIA process it was not intended as a detailed review of these procedures. Impact assessments are highly technical processes, whose success depends on the quality of the regulatory requirements, availability of analytical tools and technical capacity. Here we focused on aspects of EIA that we think influence implementation of the mitigation hierarchy. Moreover several publications have conducted broader analysis of EIA process in the region, including operational and implementation issues [27]–[31]. In addition a recent review by Reid et al. (in prep.) analyzes the SEA procedures in the region. To evaluate mitigation frameworks as the basis for offset practices, our assessment is focused on regulatory features directly related to impact mitigation, mainly: impact evaluation and the use of the mitigation hierarchy. The aim is to assess to what extent the reviewed policies set requirements that may eventually promote solid mitigation practices. This portion of our analysis serves as the starting point for the more detailed review of offset frameworks, see below.

To standardize the review as much as possible, we have defined a set of questions that have been answered for each country on a yes/no basis, depending on the contents of their laws and regulations (Appendix S2). We grouped policies for the review as follows: General (policies that apply to all projects: environmental acts, general EIA, and habitat-specific laws and regulations), and Sector-specific (mining, hydrocarbons, energy (electricity), transport infrastructure (i.e. roads, railways, airports and ports), and waste management). All the projects covered under these policies also have to follow the requirements set by general laws and regulations, so the sectorial provisions supplement the general ones.

### Review of Offset Specific Policies and Laws

Our assessment of the environmental licensing processes of the seven countries identified four (Brazil, Colombia, Mexico and Peru) that have developed specific policies that regulate offset implementation (Table 1). For these four countries we focused on the following laws that dictated offset usage:

**Table 1:** Current policies reviewed for the selected offset frameworks.

Country	Year	Document reference	What it regulates
Brazil	2000	Law 9985	Sets the obligation for projects subject to environmental licensing of offsetting impacts by making payments to support the National System of Protected Areas
	2002	Decree 4340	Regulates calculation of offset payments, sets the need of an Offsets Chamber, and establishes how to use offset funds
	2004	Direct action of unconstitutionality 3378	Partially modifies Art.36 § 1° of Law 9985 (original one declared partially unconstitutional)
	2006	CONAMA Resolution 371/06	Sets guidelines for the environmental authority to calculate, collect, use, approve and manage offset funds related to Law 9985
	2006	Decree 5746	Regulates offsets for impacts to Natural Heritage Reserves
	2009	Decree 6848	Modifies Decree 4340
	2010	Ordinance 416	Creates the Environmental Offsets Federal Chamber (CFCA)
	2010	Ordinance 458	Designates the representatives of each organization that compound the Environmental Offsets Federal Chamber (CFCA)
	2011	Ordinance 10	Regulates the selection of environmental non-governmental organizations that will be part of the Environmental Offsets Federal Chamber (CFCA)
	2011	Ordinance 225	Creates the Environmental Offsets Federal Committee (CCAF)
	2011	Normative Instruction 8	Regulates the Environmental Offsets procedure set in Decree 4340 and modified by Decree 6848
	2011	Normative Instruction 20	Regulates the administrative procedures for setting the terms of commitment regarding offsets
	2011	IBAMA Ordinance 16	Sets the bylaws of the Environmental Offsets Federal Committee (CCAF)
Colombia	2010	Resolution 1503	Sets the obligation to follow the instructions of the “Manual for allocating offsets for loss of biodiversity” for implementing offsets in projects subject to EIA
	2012	Resolution 1517	Approves the Manual for allocating offsets for loss of biodiversity
Mexico	2003	General Law on Sustainable Forestry	Sets the obligation of making offset payments for land-use change of forest areas
	2005	Regulation of the General Law on Sustainable Forestry	Sets the basis for regulating offset payments for land-use change of forest areas
	2005	Agreement on offsets equivalency	Sets the method for calculating the required offsets area
	2011	Agreement on offsets costs	Sets the reference costs for calculating the required offset payments
Peru	2014?	Offsets law [to be passed]	Sets the basis for offsetting impacts to biodiversity in projects subject to EIA (categories II and III)

In Brazil projects subject to environmental licensing must offset their impacts on environmental assets. Impacts on Protected Areas (Law 9985 of 2000), caves (Decree 6640 of 2006) and coastal native vegetation (Decree 5300 of 2004) shall always be offset, although the environmental authority (IBAMA) may require the developer to offset any other residual impacts identified. In addition to these laws, Law 12651 of 2012 (on the Protection of Native Vegetation) regulates offsets for impacts to native vegetation, although these are not required for obtaining an environmental license so we will not examine it in this paper. In this case, our review will focus on the framework first set by Law 9985 of 2000 (see Table 1).

In Colombia projects subject to EIA must offset their impacts on terrestrial ecosystems (as regulated by Resolution 1517 of 2012) and freshwater (Law 99 of 1993, Decree 1900 of 2006 and Decree 1933 of 1994). In addition there are some offset requirements for impacts to forests (Decree 1791 of 1996) as well as several specific activities (Resolutions that implement TORs for elaborating EISs, see Table S1). However, these latter policies only address a few aspects related to offset implementation, so they cannot be considered equal to the 2012 law focused on terrestrial ecosystems. For this country we will focus the review on this 2012 framework (see Table 1).

In Mexico the Sustainable Forest Development Act of 2003 requires offsets for impacts that result in land-use change to forested areas. The recently enacted Environmental Liability Act (2013) also requires offsets, but only when impacts are not predicted or approved in the EIA and are deemed an environmental offence. Since this is not a part of the environmental licensing process we have not included it in our assessment. The general law on ecological balance and environmental protection of 1988 (most commonly known as the LGEEPA) also enables the use of offsets as does the Official Mexican Rule NOM-120-SEMARNAT-2011 which makes some provisions for offsets related to mining projects. Neither of these two laws can be considered a specific offset framework since they only enable the use of offsets but do not make any specific requirements or guidance for when or how they should be used.

In Peru a new law about to be passed requires offsets for certain projects subject to EIA, and provides details on how such measures shall be implemented. Although the law is not currently enacted, we have included it in this study since it establishes a new offset framework that is different from the other country level programs.

Offset design is a complex process that entails multiple challenges. Several principles have been outlined to guide this process, the most widespread being the ones set by the Business and Biodiversity Offsets Programme (BBOP) [32]. However, applying theoretical guidance into practice often proves difficult, as when trying to translate best practice principles into effective policy requirements. Several challenges, which may be especially tricky for policy making, have been identified and discussed in the scientific literature (see [33], [34]). We want to contribute to this discussion by evaluating how the selected policies deal with these challenges, and how the theory we know may help improving legal frameworks.

Following the approach outlined in McKenney and Kiesecker 2010 [33] and Bull et al. 2013 [34] we distilled a set of criteria that constitute main current challenges and at the same time are key to policies which seek to ensure that offsets provide the following values: (1) they provide additional replacement for unavoidable negative impacts of human activity on biodiversity, (2) they involve measurable, equivalent biodiversity losses and gains, and (3) they achieve, as a minimum, no net loss of biodiversity. Following these principles we identified twelve criteria (which include most of the ones listed by the above cited references, plus two additional ones) that we used to assess the current state of offset frameworks in our four target countries (Table 2).

**Table 2:** List of criteria used for the assessment of the reviewed offset frameworks.

Criterion	Description	Discussion and Recommendations
Offset goal	Setting a target outcome (i.e. no-net-loss) and requirements for demonstrating achievement of biodiversity goal	Offset framework should set specific measureable target goals and goals should be measured against dynamic baseline, incorporating trends. Ideally net-gain, but at least no-net-loss, of biodiversity should be required [32]
Thresholds	Requirements to determine threshold for which biodiversity offset are not acceptable	Offset frameworks should acknowledge there are things that cannot be offset and thus define criteria for when the use offsets is not appropriate and avoidance or minimization should be applied [32]. These criteria could include the irreplaceability of biological resources or the irreversibility of the impacts [34]
Offset currency	Metrics for measuring biodiversity	Offset valuation should use multiple or compound metrics and incorporate measure of ecological function as well as biodiversity [34]
Equivalence	Requiring equivalence between biodiversity losses and gains	Offset should not allow ‘out of kind’ trading unless this involves ‘trading up’ from losses that have little or no conservation value. Adherence to the “like-for-like or better” principle is recommended [10]
Offset timing	Deciding in which moment offsets should be implemented	Ideally, offsets should be implemented in advance of the project so that their benefits are already in place when impacts occur [51], [68]
Time lag	Deciding whether an additional offset for the temporal loss is required in case there is a temporal gap between impact & offset gains	There is no way of completely offsetting the possible negative consequences of time lags. However, where offset benefits cannot be delivered prior to impacts it is often recommended that offset value should be discounted to account for temporal loss [56]
Offset longevity	Deciding how long offset schemes should endure	Offsets should last at least as long as the impacts of development and should be adaptively managed for change. Ideally, they should be permanent [32], [33]
Uncertainty	Establishing requirements for managing for uncertainties throughout the offset process	Uncertainty may be avoided by implementing offsets in advance. When this proves not feasible increasing offset ratios may minimize uncertainty over offset gains, although the effectiveness of this approach is still being discussed ([51]
Additionality	Ensuring that offset actions result in additional conservation outcomes that would not have occurred without the use of an offset	Ideally all offset actions should seek to provide additionality [32]. Policies should require project developers to demonstrate the gains achieved through offsets.
Link to landscape-level conservation goals	Ensuring offsets benefit broader landscape level conservation goals	Offsets should seek to complement landscape level conservation goals [42]
Offset monitoring	Requiring post implementation monitoring to track progress of projected offset benefits	Offset frameworks should always seek to monitor projected returns for a period long enough to ensure the offset values have reached maturity

## Results

### Policies Related to Application of the Mitigation Hierarchy

**Figure 2:**
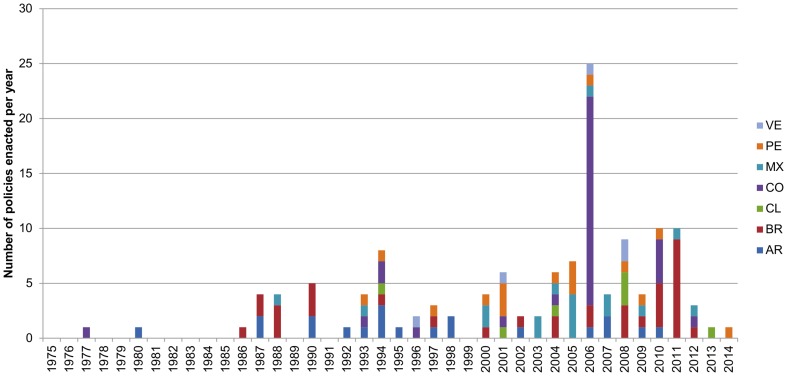
Timeline of the policies included in the study. The graphic represents the number of policies related to the environmental licensing system enacted per year on each of the studied countries. Revoked policies have not been included. Countries' names have been abbreviated to the codes set by ISO 3166.

All the surveyed countries have national-level EIA laws or regulations that cover all the habitats present in their territories. In addition some have also developed specific EIA or environmental management regulations for particular types of development, e.g. energy and mining (see Table S1). As Figure 2 shows, most environmental policies related to licensing processes in the reviewed Latin-American countries have been enacted in the last ten years. None of the countries have explicitly established a general goal of no-net-loss or net-gain for the EIA process. Only the general EIA regulations of Chile, Colombia and Mexico specifically mention the complete mitigation hierarchy (avoid, minimize, restore, offset) although none of them explicitly requires adherence to it.

Cumulative/Indirect Impacts and Impact Significance: Several countries make provisions for evaluating strategic development plans under their general EIA regulations (Chile, Decree 40 of 2013; Peru, Supreme Decree 019-2009-MINAM; Mexico, LGEEPA of 1988; Venezuela, Decree 1257 of 1996). Some sector-specific policies require the assessment of impacts from a landscape perspective (see Table S1), but for most the scale of impact assessment is not clearly stated. Only Brazil, Chile and Peru include provisions for assessing indirect impacts as part of their general EIA policies, although Argentina and Colombia add that requirement in some of their sectorial policies (roads and hydrocarbons, respectively). Cumulative impacts are required in all EIAs in all countries except for Argentina and Chile. Although Argentina includes assessment of cumulative impacts under Law 26331 of 2007 on native forests and some sector-specific policies. When it comes to how to evaluate impact significance Argentina, Brazil, Colombia and Peru provide some guidance, although only Colombia and Peru include that in their general EIA policies. In most cases, this guidance consists of a list of environmental assets that should be tackled in impact evaluation (e.g. soils, wildlife), or a list of impact characteristics that should be evaluated (e.g. positive/negative, medium/long term). However, more detailed guidance can be found in some sector-specific regulations, especially in the case of Argentina (see Table S2 for details).

**Figure 3:**
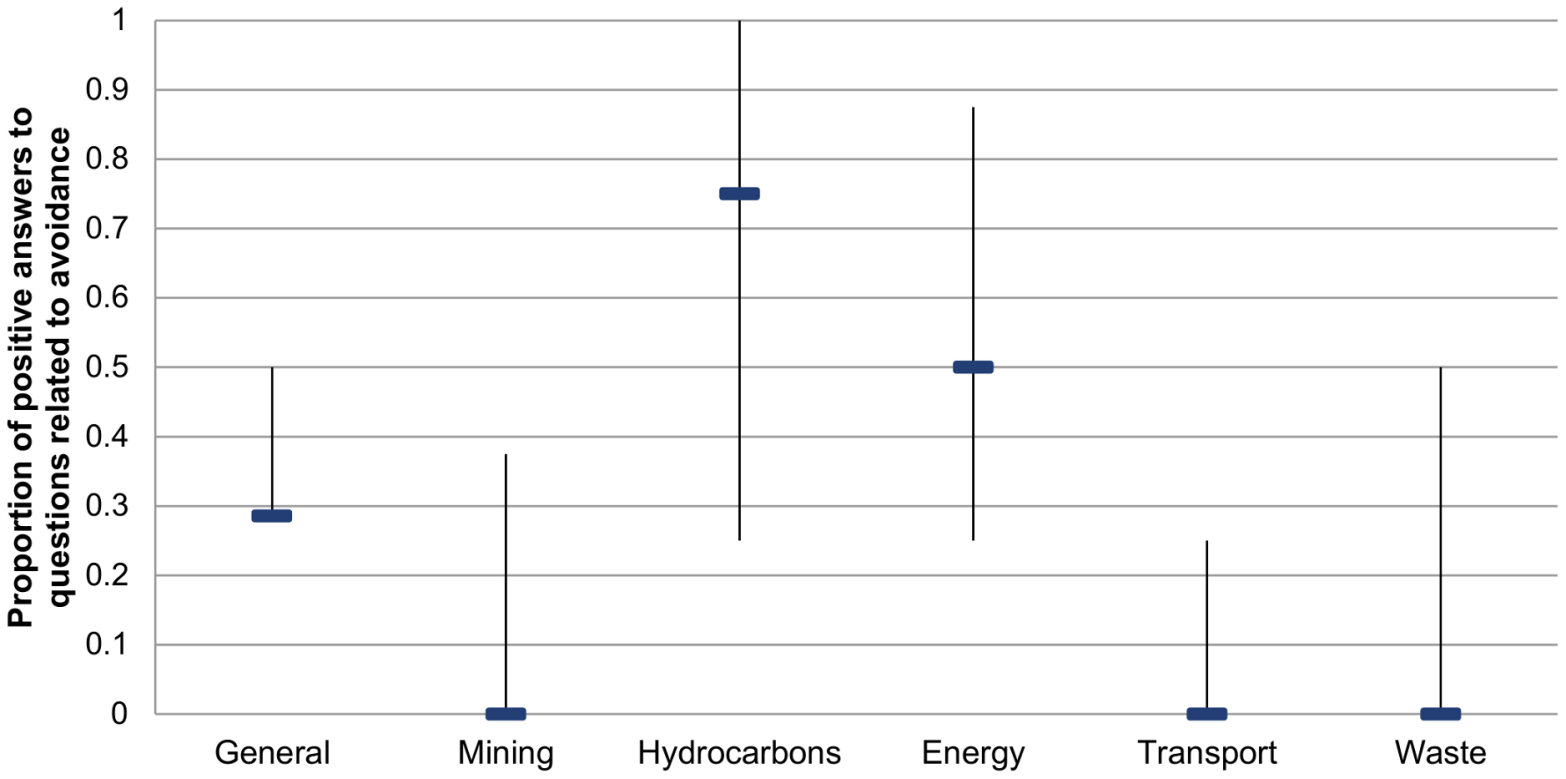
Median and standard-deviation of avoidance provisions in current sector-level policies.

Avoidance: Our results indicate that environmental licensing provisions targeted at the hydrocarbon sector have the strongest requirements for avoidance of impacts followed by provisions targeted at all energy-related development. These sectorial policies frequently include guidance and recommend activities to avoid impacts, although these requirements vary greatly among countries. The rest of sectorial policies do not seem strong regarding avoidance (Figure 3), and in some countries specific policies for certain sectors have not been found (Table S1). Only in two cases (Resolution 1604/2007 on environmental assessment and management for road projects in Argentina, and Resolution 1288 of 2006 on the TOR for EIS of electric lines in Colombia) do laws clearly state that avoidance shall be prioritized over all other forms of mitigation (See Table S3 for details). Apart from this, most provisions related to impact avoidance are found in habitat-specific or protected areas policies, which establish general thresholds for what can or cannot be done in certain habitats (such as wetlands) or in proximity to protected areas.

Minimization and Restoration: Similar to avoidance it is laws directed at the energy sector that includes the highest percentage of provisions regarding minimization and restoration. For most countries no provisions for minimization or restoration requirements are found in the other sectors. Many of the provisions that refer to minimization or restoration in the general environmental licensing process occur in reference to habitat-specific documents. Most commonly, those policies set a list of environmental assets that shall be restored if negatively impacted. While a few laws make specific recommendations for certain projects it is typically in reference to how those activities shall be carried out or establish performance standards to be met (e.g. survival rates for reforestation activities). See Table S4 for details.

Offsets: Although most countries enable the use of offsets only Brazil, Colombia, Mexico and Peru explicitly require their implementation for specific impacts. In Chile some basic provisions are established in the national EIA regulation (see Decree 40 of 2013), although more specific guidance is being developed by the Ministry of the Environment. Detailed guidance is provided by Brazil, Colombia, Mexico and Peru all of which have specific regulations regarding offsets. These countries all include regulations that are already implemented or in the case of Peru are about to be passed into law. None of these regulations is sector-specific. While Brazilian and Mexican policies are aimed at impacts to specific natural assets, Colombia and Peru have a broader scope. For more details see section on offsets below.

Monitoring: While all countries require the use of EIA and many have requirements that emphasize the use of offsets few have explicit language requiring monitoring of development impacts and mitigation activities. Some countries add provisions specific to particular sectors explicitly requiring post-project monitoring, but such information is lacking in most general-scoped EIA policies. Several of the documents that make provisions for monitoring require specific activities to be included in the plan (schedule, indicators, human resources, etc.). Some policies state when the monitoring activities should be performed (e.g. construction and closure phases), but only three documents were found to set the duration of monitoring activities, which ranged from 3 to 10 years after the completion of the project or implementation of the mitigation activities (See Table S5 for details).

### Offset Specific Review

#### Overview by country

**Figure 4:**
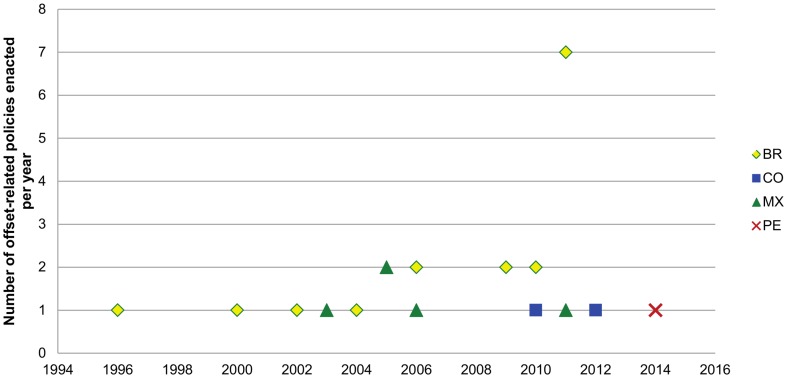
Number of policies related to each country's offset framework issued per year. Includes both current and revoked policies. Countries' names have been abbreviated to the codes set by ISO 3166.

Brazilian and Mexican schemes are the first for which specific offset policies were enacted and in turn include a relatively high number of policy documents (especially in the Brazilian case). In marked contrast the Colombian and Peruvian frameworks are recent, and have few policy related documents (See Table 1 and Figure 4). Here we consider the aspects of country-level offset policies highlighting aspects that promote conservation outcomes. Table 3 summarizes the results that are described below with more detail.

**Table 3:** Summary of results of the review of offset frameworks by country.

	Brazil	Colombia	Mexico	Peru
**Offset goal**	Balancing project impact on protected areas with equivalent gains on the SNUC	Biodiversity no-net-loss	Balancing land-use change of forests with equivalent forest gains	Biodiversity no-net-loss or net-gain
**Offset currency**	Finance-based	Area	Forest area and restoration cost	Area
**Equivalence**	Does not prioritize in-kind	In-kind	Since the money goes into a fund, equivalency is supposed but not monitored	In-kind
**Offset timing**	Payment shall be made within 10 days from the date the ToC are signed. Direct implementation by the developer shall be done within 120 days from that date (deadline can be extended)			When environmental license is approved
**Time lag**				Allows for the use of CBs to reduce losses due to time lags
**Offsets longevity**	Considered permanent, as they benefit the SNUC	For the length of the project		For the duration of impacts
**Uncertainty**				Allows for the use of CBs to reduce uncertainty
**Thresholds**		Sets exclusion areas		
**Additionality**				Requires demonstrable gains
**Link to landscape-level conservation goals**	Linked to the SNUC	Coordinates with country's conservation portfolio		Foresees coordination with national conservation priorities
**Offset monitoring**		Requires comparing results against base line		
**Transparency**	Offset projects and license applications shall be made public	A public register of offset places will be set		A public register of offset places will be set

SNUC: National System of Protected Areas in Brazil; ToC: Terms of Commitment; CBs: conservation banks.

In Brazil all projects subject to EIA can utilize offsets and those EIAs shown to cause negative impacts on protected areas must implement offsets according to the scheme set by Law 9985. The effective implementation of the offsets can be carried out either by the developer (Normative Instruction 20 of 2011, article 11) or by the agency responsible for managing the protected area (ICMBio [Chico Mendes Institute for Biodiversity Conservation] in the case of Federal Protected Areas). Offsets will always be aimed at supporting conservation units of the National System of Protected Areas (SNUC, by its Brazilian acronym; Law 9985). During the time this system has been operational, it has generated over US$200 million to be invested in protected areas (Gustavo Pinheiro, personal communication).

In Colombia offsets are required for all projects subject to EIA that cause significant impacts on terrestrial ecosystems (Resolution 1517 of 2012, second article). The developer of the project is responsible for implementing the offsets, although the location is decided by the National Environmental License Authority (ANLA) in accordance with the provisions set in the regulation [35]. The newly enacted framework provides guidance for offset design and includes a series of rules developed for selecting offset sites that meet the conservation needs of potentially impacted biological targets (i.e. size, condition, landscape context) as well as rules for impacts to offset ratio determinations based on a structured and transparent approach [36]. Offsets can either benefit the National System of Protected Areas (SINAP) or be independent of it [35], [36].

In Mexico offsets are always required for land-use change in forest areas (Ley General de Desarrollo Forestal Sustentable 2003). The agent responsible for offset implementation is the National Forest Commission (CONAFOR by its Spanish acronym) (Reglamento de la Ley General de Desarrollo Forestal Sustentable 2005), which decides the allocation of offset funds in projects implemented by different entities (agrarian communities, land owners, public administrations, research and education institutions and NGOs among others). There are no requirements for integrating offset activities into broader conservation priorities, and payment to the Mexican Forest Fund is the only tool enabled for developers to comply with the legal requirements regarding offsets (Ley General de Desarrollo Forestal Sustentable 2003). In 2013, approximately US$ 30 million have been allocated in reforestation projects related to this offset scheme [37].

In Peru most projects subject to EIA would be covered under the new law, although this is subject to the discretion of the Ministry of Environment and EIAs can be exempt from inclusion. The proposed law establishes that the developer be responsible for implementing the required offsets. Offsets are not required to be integrated into existing conservation priorities. While the law enables the developer to directly implement offsets, it also makes provisions for the creation of conservation banks.

#### Overview by Offset Criteria

Our review of the key offset criteria (Offset Goal, Offset Currency, Equivalence, Offset Timing, Time lag, Offset longevity, Uncertainty, Thresholds, Additionality, Linking offsets to Landscape-level conservation goals, Monitoring) suggests that relative to the idealized form of the regulations there are both situations when criteria appear to conform and many opportunities where regulations can be improved.

Offset Goal: not all the reviewed frameworks explicitly state the objective of compensatory mitigation, and only Peru and Colombia set no-net-loss and net-gain of biodiversity as goals for offsets. To ensure they meet these goals it will be necessary to include a framework to adjust impact to offsets ratios (see “Offset currency” subsection below).

Offset Currency: acreage seems to be the most common currency for calculating the equivalence between impacts and offsets. None of the reviewed Latin-American frameworks incorporates ecological function (e.g. carbon storage, water purification) of either the impacted sites or of the offset sites as part of the valuation process. In the case of Colombia, a set of acreage ratios (from 1∶4 to 1∶10) has been developed according to the national significance of the impacted ecosystems [36]. Regulatory guidance on this issue has yet to be developed for Peru. While not driven by a goal of no-net-loss in Mexico, the Agreement of 2005 establishes a set of acreage ratios (from 1∶1.3 to 1∶6) that are calculated according to eight criteria: ecosystem type, degree of conservation, presence of endangered or threatened species, affected ecosystem services, proximity to protected areas, project characteristics ( = how its design affects the area), degree to which soil and vegetation resources are affected, and benefits the project will bring to the area (environmental, or social). These ratios are used to calculate the payment that the developer must make to the Forest Fund (Agreement of 2011).

Equivalence: in-kind offsets are explicitly prioritized in the Colombian and Peruvian frameworks, and each country uses a different method for calculating the equivalency between impacts and offsets. In the case of Colombia, offsets are required to match impacted ecosystems [36]. The new law in Peru does not include a detailed calculation system but defines a list of variables (e.g. type of habitat impacted, priority areas for conservation and ecosystem services,) that will have to be considered when selecting area to be used for offsets(see Annex II of the Law). The Brazilian approach includes calculations of how much money developers shall put towards offsets depending on the significance of the impacts resulting from the development activities (Decree 4340 of 2002, Decree 6848 of 2009), but there is no way of assessing the equivalence between impacted assets and offset measures.

Offset Timing: provisions regarding when offsets are implemented are not present in all the reviewed frameworks, and when they are they tend to be somewhat ambiguous. In Brazil, time limits for proving offset implementation or payment are conditioned to the signed terms of commitment. But that date is not clear so there is no way of knowing if offsets are implemented before or after the project impacts occur. In Peru offsets shall be implemented when environmental license is approved, which probably means before the project impacts occur, although this is not completely clear.

Time lag: none of the reviewed Latin-American frameworks includes provisions regarding time lags, although in the case of Peru the implementation of a national network of conservation banks, where offsets credits are generated before impacts are incurred, could help address this issue.

Offsets longevity: both Colombia and Peru clearly state the minimum duration of offsets. In Colombia offsets should last at least for the length of the project, while in Peru they are required to match the duration of the impacts. Although the topic is not specifically addressed in Brazil, benefits can be considered permanent since they benefit the National System of Protected Areas.

Uncertainty: none of the reviewed Latin-American offset frameworks includes provisions regarding uncertainty, although in the case of Peru the implementation of a national network of conservation banks would help address this issue.

Thresholds: only Colombia has set clear limits as to what can be offset.

Additionality: only Peru explicitly requires demonstrable gains that ensure that the offsetting process results in additional conservation outcomes.

Linking offsets to landscape-level conservation goals: Colombia is the country that clearly establishes the link between offsets and broader conservation plans, although Brazil also requires measures to benefit the Protected Areas System. Peru includes some broad guidance on this issue, although specifics have not been set yet, probably because they will be developed in forthcoming regulatory guidance.

Monitoring: provisions regarding post-implementation monitoring of offsets are scarce, and only Colombia requires results to be compared against the area ecological baseline. In Mexico, the CONAFOR is responsible for supervising offset project implementation and that they meet the agreed terms. None of the frameworks establishes specific time requirements for monitoring.

## Discussion

Previous studies by Tanaka in 2010 [38] and The Biodiversity Consultancy in 2013 [39] identified 56 countries in the world that have or are developing national legislation or policies around offsets. In Latin America five territories required the use of offsets (Brazil, Colombia, French Guiana, Mexico and Paraguay), nine enabled the use of this tool (Argentina, Bolivia, Costa Rica, Cuba, Guyana, Honduras, Nicaragua, Panama, Uruguay and Venezuela), and three were developing policies related to offsets (Bahamas, Chile, Belize and Peru).

Our review of environmental licensing and offset policy frameworks in Argentina, Brazil, Chile, Colombia, Mexico, Peru and Venezuela shows that these systems have been evolving recently (see Figure 2), although not yet adequate in all cases. We have found significant variation on how EIAs are utilized, the importance of adhering to the mitigation hierarchy, offset goals and approaches for addressing key challenges to implementing offsets. Despite this divergence most countries have a sound foundation from which to develop policy for biodiversity offsets, but several issues require further guidance, including how best to: (1) ensure conformance with the mitigation hierarchy; (2) identify the most environmentally preferable offsets within a landscape context; (3) determine appropriate mitigation replacement ratios to ensure that biodiversity losses and gains are equivalent; and (4) ensure appropriate time and effort is given to monitor offsets performance.

### EIA frameworks

The ability of an offset framework to deliver conservation outcomes for biodiversity depends heavily on the existence of a strong Environmental Impact Assessment process. This is because the EIA process is key to ensure that all significant impacts to biodiversity are accounted for and balanced through the application of the mitigation hierarchy [40]. When the EIA process is weak or lacking, offsets may fail to deliver potential value. Our review revealed several important flaws in the EIA process. For example, some of the surveyed countries do not require all EIAs to consider indirect impacts. Indirect impacts are impacts on the environment, which are not a direct result of the project, often produced away from or as a result of a complex pathway. In the case of building a new road, for example, they not only include environmental pressure exerted by the road itself (impacts on vegetation, wildlife and the physical environment etc.), but also the land occupied by producers of road construction materials e.g. mining operations providing the road base materials. These impacts are generally off-site, and may even occur a great distance away from the direct impacts of development. But failure to consider indirect impacts underestimates environmental impacts and can obviously undermine any attempt to achieve a goal of no-net-loss [41]. Most countries also fail to incorporate the mitigation hierarchy as part of the EIA process. Grounding decisions squarely in the mitigation hierarchy will ensure that offset usage conforms to necessary conservation outcomes [42], [43]. Only the EIA regulations of Chile, Colombia and Mexico properly reference the complete mitigation hierarchy (avoid, minimize, restore, offset) although none of them explicitly requires adherence to it.

### Mitigation hierarchy

Offset frameworks clearly need to emphasize the importance of the mitigation hierarchy—avoiding and minimizing/restoring impacts before proceeding to compensatory mitigation—without reference to the hierarchy in the EIA process there is little opportunity to ensure projects conform to it. Most guidance tends to focus on avoiding impacts to “difficult-to-replace” and “high significance” resources, but ultimately provides wide discretion to regulatory authorities on decisions about when to avoid, minimize, or offset [33], [42], [44]. Our review has shown that while advancing quite detailed offset policies, countries do not seem to have strong requirements regarding impact avoidance. Avoidance requirements found in environmental licensing policies were not very strong according to our survey (see Figure 3), and it has only been found to be explicitly required in two of the offset frameworks reviewed (Colombia and Peru). Several authors have suggested that if not implemented according to the mitigation hierarchy and a set of standards, the expanded use of biodiversity offsets could provide a “license to trash”, allowing development in areas where impacts should have been avoided or more effectively minimized [33], [45], [46]. We propose that the strengthening of avoidance requirements in mitigation frameworks will require the inclusion of explicit statements requiring adherence to the mitigation hierarchy and prioritization of avoidance measures in policies related to the environmental licensing process (both general and sectorial). Additionally, offset policies should also address this issue from the perspective of avoiding impacts on elements that cannot be replaced or for impacts that are themselves irreversible [32], [34], [47]. Such guidance should focus on common species as well as rare and species at risk of imminent extinction as proposed by Regnery et al. [48]. Guidance should also incorporate science-based criteria, irreplaceability and vulnerability, examined through a systematic conservation planning framework as put forward by Kiesecker et al. [42]. Latin American countries are not alone in their lack of strong policy and regulation related to avoidance. There is broad agreement among scholars, scientists, policymakers, and regulators that in most mitigation frameworks the first and most important step in the mitigation hierarchy, avoidance, is ignored more often than it is implemented [33], [44].

### The No Net Loss goal

Offsets are intended as the last option for addressing environmental impacts of development after efforts have been undertaken to minimize impacts on-site through application of the other steps of the mitigation hierarchy: avoid, minimize, restore [6]. They seek to ensure that inevitable negative environmental impacts of development are balanced by environmental gains, with the overall aim of achieving a net neutral or positive outcome [33], [49]. As a goal, no-net-loss or net-positive-impact provides a benchmark against which the scope and effectiveness of mitigation actions can be measured. Without a goal, mitigation is simply a collection of actions; there is no clear basis for assessing which actions are more important to take (to achieve what?) or how much is enough. Impact and offset accounting will matter greatly in evaluating a project's progress toward its goal. It is worth noting that no-net-loss accounting is not an entirely new frontier: the principles underpinning mitigation accounting are similar to those developed for greenhouse gas emissions accounting (see for example “net positive climate impacts” [50]). The goal of a mitigation framework should be the first thing to be clearly set by the policies that regulate it [51]. Our results show that most mitigation policies do not define their environmental goals, and only in the cases of Colombia and Peru are these goals clearly defined in their offset policies. This lack of information about policy goals has also been noted for other countries and other environmental regulations [52]. This is a major subject to be addressed in future policy development. Only when goals are clearly defined can mitigation measures be properly designed, and progress evaluated.

### Offsets timing and habitat banks

While there remain many offset accounting challenges that need to be addressed e.g. timing and permanence of offsets, significant progress is being made driven by science and practice [53]–[55]. One of the most effective ways of avoiding these problems is to implement offsets in advance so that they deliver conservation benefits before the impacts occur. However, provisions regarding when offsets shall be implemented are not present in many of the reviewed frameworks, and when they are, they are not clearly stated. Adding a clear requirement for implementing offsets in advance of project impacts should be a priority for future policy updates in all the countries reviewed. However, impact prediction may not be accurate, and offsets that were implemented in advance may have to be adjusted as real impacts are evaluated in the field. Adaptive management will play a key role in this regard (see subsection about monitoring). From the business perspective, delivering offset benefits before impacts occur may be impractical under some circumstances, as they require long time to be fully established. Business objectives are also subject to change as markets fluctuate making detailed development plans challenging to assess proactively. But where offsets are implemented after project work begins it will be important to minimize losses due to time lags. Sometimes the use of multipliers (e.g. increasing the size of the offset) has been proposed to balance the losses due to time lags [56], [57]. However, recent research suggests that this approach does not guarantee against the shortages triggered by temporal delays that can threaten the achievement of meaningful offset gains [54], [55]. Habitat banks (also called ‘biodiversity banks’, ‘conservation banks’ or, in the US, ‘mitigation banks’) may help reduce uncertainty and the need to consider time lags because they provide the opportunity to implement anticipated offsets: by the time a credit is bought the offset activities it accounts for have long been implemented. Habitat banks also provide advantages to on-site and small parcel mitigation. By consolidating necessary services to create, maintain, and monitor, habitat banks are able to provide services at a lower cost [51], [58]. Because habitat bank credits are created prior to impacts, purchasing credits from a habitat bank decreases permitting time [59]. The cost of achieving a certain level of performance and duration is often lower than other offsets options and regulatory burden and risk is passed from developer to habitat bank. We propose that habitat banking can help implement offsets and provide positive conservation outcomes that may not have been achieved otherwise [60]. For example, buying habitat banking credits is sometimes the only feasible offsetting option for small companies which have no capacity to carry out offset projects by themselves. More importantly, habitat banks aggregate multiple offset activities into few, larger projects, which are more likely to deliver conservation outcomes [61]. Such aggregation would probably be harder to achieve through other means. However, of all the reviewed countries only the new offset law in Peru allows for the use of habitat banking (called ‘conservation banking’). Incorporation of this tool into existing mitigation frameworks may improve the implementation of offsets and gains for conservation.

### Landscape scale

Historically mitigation has occurred primarily in a reactive fashion at small spatial scales on a site-by-site basis but there is general consensus among research and practitioners that mitigation should be a more comprehensive approach that considers whole systems, anticipates impacts, and recommends effective actions to keep our natural systems healthy [42]. Integrating mitigation at a landscape scale moves beyond a project-by-project approach to one that can support a dynamic vision consistent with broader conservation goals. A landscape vision is essential because it ensures that the biologically and ecologically important features remain essential throughout the process. Without this vision, the sight of the overarching conservation targets is lost, establishing priorities becomes difficult, and limited resources may be squandered. In this sense, the Colombian and Peruvian offset frameworks are progressive, as they have been developed from a landscape conservation perspective. These frameworks also require offsets for impacts to all natural ecological systems. Compare this to the use of offsets in the United States, where offsets are typically only used to address impacts to wetlands and for threatened species. These new frameworks in Colombia and Peru can serve as an example to be followed by future offset policies not only in Latin America but globally.

Moving forward, we hope that offset frameworks develop guidelines that prompt practitioners to think strategically about offset site selection, and to develop practical guidelines for how to select offset sites. Site selection for offsets should be an exercise in landscape ecology. Using quantitative site selection tools [62], [63], or blending this process as part of landscape level conservation plans, to provide a transparent, flexible and rule-based approach towards guiding site selection. Moreover, if political pressures constrain practitioners to a particular political extent, quantitative site selection tools will allow them to assess if meeting goals are possible given those constraints [49]. When it comes to offsets, failure to systematically select suitable sites could reduce the potential benefits for conservation.

### Monitoring

Post-implementation monitoring should be a key component of every mitigation framework. Monitoring is a way of ensuring compliance with policy requirements, evaluating the achievement of the mitigation goals, and getting feedback on the effectiveness of the activities implemented [34], [64]. It is also the primary driver of adaptive management, a necessary procedure for getting long-term conservation outcomes [33], [46], [65]. In some way, it is also an essential component for transparency of the process, since the public does not only need to know which activities are proposed and how mitigation funds are allocated (information that many of the reviewed countries already provide for offsets), but also if and how such actions are carried out. However, the lack of post-implementation monitoring is a common problem in mitigation and conservation projects in general [44], [66]. Even when follow-up programs are required, they are often required for a short period, and because of the short temporal scale problems with offset implementation frequently go undetected [54]. Many of the countries in our survey lack provisions that guide the monitoring of impacts and mitigation measures, and the few cases that do require monitoring typically require short monitoring periods. Lack of enforcement of environmental policies related to offsets is a common problem [58], [67]. The requirement of solid monitoring processes is the first step to address these issues and will need to be key component of any mitigation policies if they are to promote sustainable development.

## Conclusion

Our results indicated that all the surveyed countries have national-level Environmental Impact Assessment laws or regulations and most enable the use of offsets but only Brazil, Colombia, Mexico and Peru explicitly require their implementation. While several countries may have quite detailed offset policies, most countries do not seem to have strong requirements regarding impact avoidance which could undermine the use of offsets. While the most recent frameworks (those from Colombia and Peru) show more adherence to the theoretical recommendations we outlined there are still some principles that have not been included in most country level frameworks. In some cases, this may be due to the lack of scientific agreement on how to address certain issues in practice. To ensure that the use of offsets advances biodiversity conservation going forward it will be necessary to develop further guidance on how best to: (1) ensure conformance with the mitigation hierarchy; (2) identify the most environmentally preferable offsets within a landscape context; (3) determine appropriate mitigation replacement ratios; and (4) ensure appropriate time and effort is given to monitor offsets performance. Despite these shortcomings most countries have a strong foundation from which to develop policy for biodiversity offsets. In addition to these issues the Business and Biodiversity Offsets Program, by far the largest multi-stakeholder effort to examine biodiversity offsets, stresses the importance to ensure that offsets involve stakeholder participation, the fair and equitable distribution of offsets benefits and use of traditional knowledge in offset design. While we agree these are important issues they were not included in our analysis given our focus on the theoretical scientific issues involved in offset design.

Although policies and regulatory guidance alone will not deliver conservation value without regulatory oversight and implementation capacity. The effectiveness of an offset program demands a responsible administrative entity with firm requirements for adequate oversight, performance accountability, and process transparency and fairness. Achieving these objectives requires several administrative functions, including: 1) communication and maintenance of standards and protocols; 2) application of standards to individual projects to analyze impacts and determine needs for mitigation; 3) coordination and oversight of mitigation planning to target mitigation funding toward projects with high conservation return on investment; 4) oversight of mitigation funds to ensure appropriate fiduciary management and impartial allocation; 5) a process that utilizes monitoring and provides a mechanism to adjust activities based on monitoring results; and 6) procedures for sanctions against failure to achieve legal requirements to make sure that laws are effectively implemented. An independent third-party entity that oversees these functions will be essential.

## Supporting Information

Table S1 **Reviewed policies (listed by country and by sector) that have provision related to mitigation.**
(DOCX)

Table S2 **Guidance for environmental impact assessment provided in the reviewed policies.**
(DOCX)

Table S3 **Guidance for impact avoidance found in the reviewed policies.**
(DOCX)

Table S4 **Guidance for impact minimization and restoration found in the reviewed policies.**
(DOCX)

Table S5 **Guidance for monitoring activities found in the reviewed policies.**
(DOCX)

Appendix S1 **List of sources.**
(DOCX)

Appendix S2 **Questions used to assess country level environmental impacts assessment process.**
(DOCX)
