# Supplementary material for: Policy Development for Environmental Licensing and Biodiversity Offsets in Latin America
Source: PLoS One. 2014 Sep 5;9(9):e107144. doi: 10.1371/journal.pone.0107144 (PMC4156437; doi:10.1371/journal.pone.0107144)
Supplement: Table S1 — Reviewed policies (listed by country and by sector) that have provision related to mitigation. (DOCX) [file pone.0107144.s001.docx]

Table S1. Reviewed policies (listed by country and by sector) that have provision related to mitigation.

| **GENERAL SCOPE (NOT SECTOR-SPECIFIC)** | | | |
| --- | --- | --- | --- |
| **Country** | **Year** | **Document reference** | **Subject** |
| Argentina | 1980 | Law 22351 | National Protected Areas |
|  | 1990 | Decree 2148/1990 | Strict Natural Reserves |
|  | 1994 | Decree 453/1994 | Natural reserves |
|  | 1994 | Law 24354 | Public investments |
|  | 1994 | Resolution 16/94* | Regulation of EIA in National Parks |
|  | 1995 | Resolution 501/95 | General environmental guide for investment projects |
|  | 2002 | Law 25675 | Environmental Act |
|  | 2007 | Law 26331 | Native forests |
|  | 2009 | Decree 91/2009 | Environmental protection of native forests |
|  | 2010 | Law 26639* | Glacier preservation |
| Brazil | 1986 | CONAMA Resolution 001/86 | General directions on EIA |
|  | 1987 | CONAMA Resolution 010/87 | Requisites for environmental licensing of large projects |
|  | 1988 | Constitution of Brazil | |
|  | 1988 | Law 7661 | National Plan of Coastal Management |
|  | 1990 | CONAMA Resolution 013/90 | EIA for projects close to Conservation Units |
|  | 1997 | CONAMA Resolution 237/97 | Projects subject to environmental licensing |
|  | 2000 | Law 9985 | Protected Areas |
|  | 2002 | Decree 4340 | Regulation of environmental conservation |
|  | 2004 | Decree 5300 | Regulation of coastal management |
|  | 2004 | Direct action of unconstitutionality 3378 | Partially modifies Law 9985 [offsets] |
|  | 2006 | CONAMA Resolution 371/06 | Guidelines on the use of offset funds |
|  | 2006 | Decree 5476 | Regulation of National System of Nature Conservation Units |
|  | 2008 | Decree 6640 | Protection of caves |
|  | 2008 | IBAMA Normative Instruction 184/2008 | Regulation of EIA administrative process |
|  | 2009 | Decree 6848 | Regulation of environmental offsets |
|  | 2010 | IBAMA Normative Instruction 12/2010 | Offsetting of greenhouse emissions |
|  | 2010 | Ordinance 416 | Regulation of environmental offsets |
|  | 2010 | Ordinance 458 | Regulation of environmental offsets |
|  | 2011 | Ordinance 10 | Regulation of environmental offsets |
|  | 2011 | Ordinance 225 | Regulation of environmental offsets |
|  | 2011 | Normative Instruction 01/2011 | Restricted areas for development |
|  | 2011 | Normative Instruction 20/2011 | Implementation of offsets |
|  | 2011 | Normative Instruction 8/2011 | Regulation of environmental offsets |
|  | 2011 | IBAMA Ordinance 16 | Regulation of environmental offsets |
| Chile | 1931 | Decree 4363 | Forests Act |
|  | 1994 | Law 19300* | Environmental Act |
|  | 2004 | Decree 238 | Regulation of marine parks and reserves |
|  | 2008 | Decree 93 | Regulation of the restoration of native forests and forestry development |
|  | 2008 | Law 20283 | Restoration of native forests and forestry development |
|  | 2008 | Law 20293 | Protection of cetaceans |
|  | 2013 | Decree 40* | EIA Regulation |
| Colombia | 1974 | Decree-Law 2811 | Natural resources |
|  | 1977 | Decree 622 | Partial regulation of Decree-Law 2811 on the National Park System |
|  | 1991 | National Political Constitution | |
|  | 1993 | Law 99 | Creation of the Ministry of the Environment |
|  | 1996 | Decree 1791 | Regulation of forestry |
|  | 2004 | Resolution 157 | Regulation of the use, conservation and management of wetlands |
|  | 2006 | Decree 1900 | Regulation of art. 43 of Law 99 [freshwater] |
|  | 2006 | Resolution 1255* | TOR for evaluation of alternatives in non-linear projects |
|  | 2006 | Resolution 1277* | TOR for evaluation of alternatives in linear projects |
|  | 2010 | Decree 2372 | National System of Protected Areas |
|  | 2010 | Decree 2820 | Environmental licensing regulation |
|  | 2010 | Resolution 1503 | General methodology for elaborating EISs |
|  | 2011 | Law 1450 | National Development Plan 2010-2014 |
|  | 2012 | Resolution 1517 | Biodiversity offsets manual |
| Mexico | 1988 | LGEEPA* | General Law on Ecological Balance and Environmental Protection |
|  | 1993 | General Law on Human Settlements | |
|  | 2000 | General Law on Wildlife | |
|  | 2000 | Regulation of the general law of the environment regarding EIA* | |
|  | 2003 | General Law on Sustainable Forest Development | |
|  | 2003 | NOM-022-SEMARNAT-2003 | Preservation, sustainable use and restoration of coastal wetlands and mangroves |
|  | 2005 | Regulation of the General Law of Sustainable Forest Development | |
|  | 2005 | Agreement for establishing offsets area equivalency | |
|  | 2011 | Agreement for establishing offsets economic equivalency | |
|  | 2013 | Environmental Liability Act | |
| Peru | 1997 | Law 26834 | Protected Natural Areas |
|  | 2000 | Law 27308 | Forests and Wildlife |
|  | 2001 | Law 27446* | EIA |
|  | 2001 | Supreme Decree 014-2001-AG* | Regulation of forests and wildlife |
|  | 2001 | Supreme Decree 038-2001-AG | Regulation of Protected Natural Areas |
|  | 2005 | Law 28611 | Environmental Act |
|  | 2009 | Supreme Decree 019-2009-MINAM* | Regulation of EIA |
|  | 2010 | Supreme Decree 001-2010-AG | Regulation of water resources |
|  | 2014 | Offsets Law [to be passed] | |
| Venezuela | 1996 | Decree 1257* | EIA regulation |
|  | 2001 | Decree-Law for coastal areas | |
|  | 2006 | Environmental Act | |
|  | 2008 | Biodiversity Management Act | |
|  | 2008 | Decree 5999 | Tourism |
| **SECTOR-SPECIFIC: MINING** | | | |
| **Country** | **Year** | **Document reference** | **Title (abbreviated and translated into English)** |
| Argentina | 1997 | Decree 456 | Mining Code |
| Brazil | 1988 | CONAMA Resolution 008/1988 | EIA for mining projects [extraction] |
|  | 1990 | CONAMA Resolution 009/1990 | EIA for mining extractive activities |
|  | 1990 | CONAMA Resolution 010/90 | Environmental licensing for class II mining projects |
| Colombia | 2001 | Law 685 | Mining Code |
| Mexico | 2005 | NOM-116-SEMARNAT-2005 | Environmental protection for seismologic prospection [wastelands] |
|  | 2012 | NOM-120-SEMARNAT-2011 | Environmental protection for mining exploration activities |
| Peru | 1993 | Supreme Decree 016-93-EM | Environmental protection for mining activities |
|  | 2005 | Supreme Decree 033-2005-EM | Regulation of mining closures |
|  | 2005 | Supreme Decree 059-2005-EM | Regulation of mining environmental stock |
|  | 2008 | Supreme Decree 020-2008-EM | Environmental rules for mining exploration activities |
| **SECTOR-SPECIFIC: HYDROCARBONS** | | | |
| **Country** | **Year** | **Document reference** | **Title (abbreviated and translated into English)** |
| Argentina | 1992 | Resolution 105/92 | Environmental protection for hydrocarbon extraction activities |
|  | 1993 | Resolution 252/93 | Guidance for EISs of hydrocarbon projects |
|  | 2006 | Disposition 123/06 | Environmental protection rules for hydrocarbon transportation |
| Brazil | 1994 | CONAMA Resolution 23/94 | Oil and gas EIA |
|  | 2011 | Normative Instruction 02/2011 | Restricted areas for oil and gas prospective activities |
|  | 2011 | Ordinance 422/2011 | Marine oil and gas EIA |
|  | 2012 | Interministerial ordinance 198* | Oil and gas developments |
| Colombia | 2006 | Resolution 1253* | TOR for EIS of hydrocarbon stations |
|  | 2006 | Resolution 1275* | TOR for EIS of hydrocarbon conducts |
|  | 2006 | Resolution 1269* | TOR for EIS of refineries |
|  | 2010 | Resolution 1543 | TOR for EIS for hydrocarbon exploitation projects |
|  | 2010 | Resolution 1544 | TOR for hydrocarbon exploration projects |
| Mexico | 2004 | NOM-115-SEMARNAT-2003 | Environmental protection for land oil drilling activities [wastelands] |
|  | 2007 | NOM-149-SEMARNAT-2006 | Environmental protection for marine oil extraction activities [wastelands] |
|  | 2009 | NOM-117-SEMARNAT-2006 | Environmental protection for oil transportation infrastructures [wastelands] |
| Peru | 2006 | Supreme Decree 015-2006-EM | Environmental protection for activities related to hydrocarbons |
| **SECTOR-SPECIFIC: ENERGY (ELECTRICITY)** | | | |
| **Country** | **Year** | **Document reference** | **Title (abbreviated and translated into English)** |
| Argentina | 1987 | Resolution SE 475/87 | EIA for energy projects |
|  | 1987 | Resolution SE 718/87 | Manual of environmental management of hydroelectric projects |
|  | 1990 | Law 23879 | EIA of hydroelectric projects |
|  | 1998 | Resolution 1725/1998 | Regulation of EISs in energy projects |
|  | 1998 | Resolution 77/1998 | Environmental management of electricity transportation |
| Brazil | 1987 | CONAMA Resolution 006 | Environmental licensing for energy projects |
|  | 2011 | Ordinance 421/2011* | EIS for electrical lines |
| Colombia | 1994 | Decree 1933 | Offset payments related to energy projects that use freshwater resources |
|  | 2006 | Resolution 1280* | TOR for EIS of hydroelectric projects |
|  | 2006 | Resolution 1284* | TOR for EIS of dams and reservoirs |
|  | 2006 | Resolution 1287* | TOR for EIS of thermoelectric projects |
|  | 2006 | Resolution 1288 | TOR for EIS of electric lines |
| Mexico | 2007 | NOM-150-SEMARNAT-2006 | Environmental protection for geothermic activities |
| Peru | 1994 | Supreme Decree 29-94-EM | Environmental protection for electricity-related projects |
| **SECTOR-SPECIFIC: TRANSPORT INFRASTRUCTURE** | | | |
| **Country** | **Year** | **Document reference** | **Title (abbreviated and translated into English)** |
| Argentina | 2007 | Resolution 1604/2007 | Manual for environmental management of road projects |
| Brazil | 2004 | CONAMA Resolution 349/2004 | EIA for small railway projects |
|  | 2010 | IBAMA Normative Instruction 02/2010 | EIA for roads |
| Colombia | 2006 | Resolution 1271 | TOR for EIS of railways |
|  | 2006 | Resolution 1272* | TOR for dredging of accesses to marine ports |
|  | 2006 | Resolution 1273* | TOR for dredging navigable channels |
|  | 2006 | Resolution 1276* | TOR for EIS of airports |
|  | 2006 | Resolution 1281* | TOR for marine ports |
|  | 2006 | Resolution 1283* | TOR for tunnels |
|  | 2006 | Resolution 1289 | TOR for EIS of road projects |
|  | 2006 | Resolution 1290* | TOR for river ports |
|  | 2006 | Resolution 1559 | TOR for EIS of second roads |
| Peru | 2007 | Ministerial Resolution 1079-2007-MTC/02 | Guidelines for EIS of transport infrastructure projects |
| **SECTOR-SPECIFIC: WASTE MANAGEMENT** | | | |
| **Country** | **Year** | **Document reference** | **Title (abbreviated and translated into English)** |
| Brazil | 2008 | CONAMA Resolution 404/2008 | EIA for waste management |
| Colombia | 1994 | Resolution 541 | Waste management |
|  | 2006 | Resolution 1274 | TOR for EIS of landfills |
| Mexico | 2003 | NOM-055-SEMARNAT-2003 | Environmental protection for dangerous waste management |
|  | 2003 | NOM-083-SEMARNAT-2003 | Environmental protection for landfills |
|  | 2006 | Regulation of the General Law on waste prevention and management | |
| Peru | 2004 | Supreme Decree 057-2004-PCM | Regulation of waste management |

* Asterisks indicate policies that require the assessment of impacts at a landscape scale (see “Results” section).
