# Supplementary material for: Policy Development for Environmental Licensing and Biodiversity Offsets in Latin America
Source: PLoS One. 2014 Sep 5;9(9):e107144. doi: 10.1371/journal.pone.0107144 (PMC4156437; doi:10.1371/journal.pone.0107144)
Supplement: Table S2 — Guidance for environmental impact assessment provided in the reviewed policies. (DOCX) [file pone.0107144.s002.docx]

Table S2. Guidance for environmental impact assessment provided in the reviewed policies

| **Country** | **Kind of policy** | **Document** | **Section** | **Kind of guidance** |
| --- | --- | --- | --- | --- |
| Argentina | Habitat-specific | Law 26331 | Art.22 | Definition of significant impact  List of impact characteristics |
|  | Protected Areas | Law 16/94 | Art.18.3 | List of environmental assets to evaluate  List of project stages to assess  List of impact characteristics to evaluate |
|  | Energy EIA | Res.1725/1998 | Annex 1.7.1 | Instructions to build the impact evaluation matrix |
|  | Energy EIA | Res. 077/1998 | Annex I | Detailed instructions to evaluate visual impacts |
|  | Hydrocarbons | Disp. 123/06 | Annex, 3.2 | Formula to calculate impact importance (includes guidance on how to assign values to the different factors) |
|  | Hydrocarbons | Res. 252/93 | Annex I, 3.4 | Categories for qualitative evaluation of impacts |
|  | General EIA | Res. 501/95 | Section I.3 | List of impact characteristics to evaluate  List of recommended evaluation methodologies  Basic directions on quantitative/qualitative evaluation |
|  | Roads | Res. 1604/2007 | Part A, sec.I, ch.5; Part B, sec.I, ch.1 | Description of impact characteristics  Description of evaluation methodologies |
| Brazil | Energy | Ord. 421/2011 | Annexes | General basic guidance  List of impact characteristics to evaluate |
| Chile | General EIA | Dec. 40/2013 | Art.18.f | General basic guidance  List of impact characteristics to evaluate |
| Colombia* | General EIA | Res. 1503 | Pages 21 and 22 | General basic guidance  List of impact characteristics to evaluate |
|  | Hydrocarbons | Res. 1544 | Section 5; also 2, 3 and 4 | General basic guidance List of elements for baseline study as a reference for impact evaluation  List of factors for economic evaluation |
|  | Waste | Res. 1274 | Sections 3, 4 and 5 | List of elements for baseline study as a reference for impact evaluation |
| Peru | General EIA | SD 019-2009-MINAM | Annex IV | List of environmental assets to evaluate  List of project stages to assess  List of impact characteristics to evaluate |
|  | Hydrocarbons | SD 015-2006-EM | Art.27.6.a | List of impact characteristics to evaluate  List of project activities to assess (all) |

Res.: Resolution, Disp.: Disposition, Ord.: Ordinance, SD.: Supreme Decree

* Colombia: Resolutions 1255 (general EIA), 1253, 1275, 1269, 1543 (hydrocarbons), 1280, 1287, 1288, 1284 (energy), 1283, 1289, 1559 (roads), 1276 (airports), 1271 (railways), 1272 (marine dredging), 1281 (marine ports), 1273 (estuarine dredging), and 1290 (river docks), provide the same guidance as Resolution 1544 (in the table), in the same sections. Resolution 1277 (general EIA) provides the same guidance in sections 6, and 3, 4 and 5.
