# Supplementary material for: Policy Development for Environmental Licensing and Biodiversity Offsets in Latin America
Source: PLoS One. 2014 Sep 5;9(9):e107144. doi: 10.1371/journal.pone.0107144 (PMC4156437; doi:10.1371/journal.pone.0107144)
Supplement: Table S3 — Guidance for impact avoidance found in the reviewed policies. (DOCX) [file pone.0107144.s003.docx]

Table S3. Guidance for impact avoidance found in the reviewed policies

| **Country** | **Kind of policy** | **Document** | **Section** | **Kind of guidance** |
| --- | --- | --- | --- | --- |
| Argentina | Protected areas | Law 22351 | Art.5 | Prohibited activities in specific areas |
|  | Protected areas | Dec. 2148/90 | Art.5 | Prohibited activities in specific areas |
|  | Hydrocarbons | Res. 105/92 | Section 2.1.1 | Recommended ways of performing activities to avoid certain impacts |
|  | Protected areas | Dec. 453/94 | Art.3 and 4 | Prohibited activities in specific areas |
|  | Energy | Res. 77/1998 | Annex I | Recommended ways of performing activities to avoid certain impacts |
|  | Hydrocarbons | Disp. 123/06 | Section 3.3 | List of impacts to avoid |
|  | Forests | Law 26331 | Art.14 | Prohibited activities in specific areas |
|  | Roads | Res. 1604/2007 | Part A, Ch.6 | List of impacts to avoid  Prioritizes avoidance over all other mitigation activities |
|  | Habitat specific | Law 26639 | Art.6 | Prohibited activities in specific areas |
| Brazil | Protected areas | Law 9985 | Arts. 9.4, 18.6, 21.3, 22.1, 23.2 | Prohibited activities in specific areas |
|  | Caves | Dec. 6640 | Art.3 | Protected assets |
|  | Species-specific | NI 01/2011 | All | Prohibited activities in specific areas |
|  | Habitat-specific | NI 02/2011 | All | Prohibited activities in specific areas |
|  | Energy EIA | Ord. 421/2011 | Annexes | Description of mitigation measures  TOR for environmental management plan (Annex IV) |
| Chile | Forests | Dec. 4363 | Art.5 | Prohibited activities in specific areas |
|  | General EIA | Dec. 40/2013 | Arts.97, 98 | Description of mitigation measures  Kinds of avoidance measures |
|  | Protected areas | Dec. 238 | Art.14 | Prohibited activities in specific areas |
|  | Forests | Law 20283 | Art.8 | Prohibited activities in specific areas |
| Colombia* | Environment | Dec. 2811 | Art.104 | Prohibited activities in specific areas |
|  | Protected areas | Decree 622 | Art. 30 | Prohibited activities in specific areas |
|  | Protected areas | Decree 2372 | Art.35.2 | Prohibited activities in specific areas |
|  | Waste | Res. 541 | Art.3 | Recommended ways of performing activities to avoid certain impacts |
|  | Hydrocarbons | Res. 1544 | Sections 6 and 7 | Guidance for defining exclusion zones  Description of mitigation measures |
|  | Energy | Res. 1288 | Sections 6 and 7 | Guidance for defining exclusion zones  Description of mitigation measures  Prioritizes avoidance over all other mitigation activities |
|  | General EIA | Res. 1503 | Section 2.5 | Description of mitigation measures |
|  | Environment | Law 1450/2011 | Arts.202, 204, 207 | Prohibited activities in specific areas |
| Mexico | Environment | LGEEPA | Arts. 47BIS-I, 50, 52, 55 | Prohibited activities in specific areas |
|  | Wildlife | LGVS | Art. 60ter | Prohibited activities in specific areas |
|  | Habitat specific | NOM-022-SEMARNAT-2003 | Sections 4.1, 4.4, 4.14-21 | Prohibited activities  Recommended ways of performing activities to avoid certain impacts |
|  | Hydrocarbons | NOM-115-SEMARNAT-2003 | Sections 4.2.2, 4.2.4, 4.2.5 and 4.2.7 | Recommended ways of performing activities to avoid certain impacts |
|  | Mining | NOM-116-SEMARNAT-2005 | Sections 4.1.1 and 4.1.3 | Recommended ways of performing activities to avoid certain impacts |
|  | Forests | Forestry regulation | Art.14.I | Protected assets |
|  | Energy | NOM-150-SEMARNAT-2006 | Sections 4.2.3 and 4.2.5-7 | Recommended ways of performing activities to avoid certain impacts |
|  | Roads | NOM-117-SEMARNAT-2006 | Sections 5.1.1 and 5.1.2 | Recommended ways of performing activities to avoid certain impacts |
|  | Mining | NOM-120-SEMARNAT-2011 | Sections 4.1.4, 4.1.6 and 4.1.19 | Protected assets  Recommended ways of performing activities to avoid certain impacts |
| Peru | Energy | SD 29-94-EM | Arts. 35, 38 and 39 | List of impacts to avoid |
|  | Protected areas | Law 23864 | Art.23 | Prohibited activities in specific areas |
|  | Protected areas | SD 038-2001-AG | Arts.46.2, and 50-55 | Prohibited activities in specific areas |
|  | Waste | SD 057-2004-PCM | Art.67 | Prohibited activities in specific areas |
|  | Environment | Law 28611 | Art. 91 | Prohibited activities in specific areas |
|  | Hydrocarbons | SD 015-2006-EM | Arts.41, 65 and 83.c | Recommended ways of performing activities to avoid certain impacts |
|  | Mining | SD 020-2008-EM | Art.115 | Prohibited activities in specific areas |
|  | Habitat specific | SD 001-2010-AG | Art.115 | Prohibited activities in specific areas |
| Venezuela | Habitat specific | Dec. on coasts | Art.20 | Prohibited activities in specific areas |

Dec.: decree, Res.: resolution, Disp.: disposition, NI: Normative Instruction, NOM: Official Mexican Rule, SD: Supreme Decree

* Colombia: Resolutions 1255 (general EIA), 1253, 1275, 1269, 1543 (hydrocarbons), 1280, 1287, 1284 (energy), 1283, 1289, 1559 (roads), 1276 (airports), 1271 (railways), 1272 (marine dredging), 1281 (marine ports), 1273 (estuarine dredging), and 1290 (river docks), provide the same guidance as Resolution 1544 (in the table), in the same sections. Resolution 1277 (general EIA) provides the same guidance in sections 7 and 8.
