# Supplementary material for: Policy Development for Environmental Licensing and Biodiversity Offsets in Latin America
Source: PLoS One. 2014 Sep 5;9(9):e107144. doi: 10.1371/journal.pone.0107144 (PMC4156437; doi:10.1371/journal.pone.0107144)
Supplement: Table S4 — Guidance for impact minimization and restoration found in the reviewed policies. (DOCX) [file pone.0107144.s004.docx]

Table S4. Guidance for impact minimization and restoration found in the reviewed policies.

| **Country** | **Kind of policy** | **Document** | **Section** | **Kind of guidance** |
| --- | --- | --- | --- | --- |
| Argentina | Energy | Res. 77/1998 | Sections 4.1 and 4.2 | Recommended minimization-restoration actions for specific impacts |
|  | Hydrocarbons | Disp. 123/06 | Section 3.3 | List of impacts to minimize or restore |
|  | Roads | Res. 1604/2007 | Part A, Ch.6 and Ch.7 | Recommended minimization-restoration actions for specific impacts |
| Brazil | General EIA | CONAMA Res. 010/87 | Arts. 1 and 2 | Recommended minimization-restoration actions for specific impacts  Minimum budget required |
|  | Energy EIA | Ord. 421/2011 | Annexes | Description of mitigation measures  TOR for environmental management plan (Annex IV) |
| Chile | Environment | Law 19300 | Art.13bis | Mitigation measures not binding for the environmental license |
|  | General EIA | Decree 40 | Arts. 97, 99 | Description of mitigation measures  Sets the objectives of restoration measures  Location: same area as impacts |
| Colombia* | Waste | Res. 541 | Art. 3 | Recommended performance to minimize impacts |
|  | Hydrocarbons | Res. 1544 | Section 7 | Description of mitigation measures  List of environmental assets to include in the mitigation plan  Recommended restoration actions |
|  | Energy | Res. 1288 | Section 7 | Description of mitigation measures |
|  | General EIA | Res. 1503 | Section 2.5 | Description of mitigation measures |
| Mexico | Habitat specific | NOM-022-SEMARNAT-2003 | Sections 4.15, 1.32, 4.37 and 4.39 | Recommended activities to minimize impacts  Rules for carrying out certain restoration activities |
|  | Forests | Forestry reg. | Art. 14.III | Location: criteria to select areas |
|  | Hydrocarbons | NOM-115-SEMARNAT-2003 | Sections 4.4.1 to 4.4.5 | Required restoration actions |
|  | Mining | NOM-116-SEMARNAT-2005 | Sections 4.2.1, 4.2.3 and 4.2.4 | Required restoration actions |
|  | Waste | Waste reg. | Chapter III | Rules for carrying out certain restoration activities |
|  | Energy | NOM-150-SEMARNAT-2006 | Sections 4.2.4, 4.2.10, and 4.5.4 to 4.5.6 | Recommended performance to minimize impacts  Rules for carrying out certain restoration activities  List of environmental assets to include in the mitigation plan |
|  | Hydrocarbons | NOM-149-SEMARNAT-2006 | Section 5.4.1 | Required restoration actions |
|  | Roads | NOM-117-SEMARNAT-2006 | Section 5.4.1 | Required restoration actions |
|  | Mining | NOM-120-SEMARNAT-2011 | Sections 4.1.18, 4.1.19, and 4.2.2 to 4.3 | Rules for carrying out certain restoration activities  List of activities to include in the restoration plan |
| Peru | Mining | SD 016-93-EM | Arts.10, 12, 13 and 27 | List of impacts to minimize or restore  Required restoration actions |
|  | Energy | SD 29-94-EM | Arts.24 and 35 | List of impacts to minimize or restore |
|  | Waste | SD 057-2004-PCM | Art.92 | Recommended restoration actions  General guidance on the design of a restoration plan |

Dec.: decree, Res.: resolution, Disp.: disposition, NI: Normative Instruction, NOM: Official Mexican Rule, SD: Supreme Decree

* Colombia: Resolutions 1255 (general EIA), 1253, 1275, 1543 (hydrocarbons), 1280, 1287, 1284 (energy), 1283, 1289, 1559 (roads), 1281 (marine ports), 1273 (estuarine dredging), and 1290 (river docks), provide the same guidance as Resolution 1544 (in the table), in the same sections. Resolution 1277 (general EIA) provides the same guidance in section 8.

Resolutions 1276 (airports), 1271 (railways), 1272 (marine dredging), 1269 (hydrocarbons), provide the same guidance as Resolution 1288 (in the table), in the same section.
