# Supplementary material for: Policy Development for Environmental Licensing and Biodiversity Offsets in Latin America
Source: PLoS One. 2014 Sep 5;9(9):e107144. doi: 10.1371/journal.pone.0107144 (PMC4156437; doi:10.1371/journal.pone.0107144)
Supplement: Table S5 — Guidance for monitoring activities found in the reviewed policies. (DOCX) [file pone.0107144.s005.docx]

Table S5. Guidance for monitoring activities found in the reviewed policies.

| **GENERAL PROVISIONS** | | | | |
| --- | --- | --- | --- | --- |
| **Country** | **Kind of policy** | **Document** | **Section** | **Kind of guidance** |
| Argentina | Protected Areas | Res. 16/94 | Art.18.6.a | Goals of monitoring activities |
|  | Environment | Res. 501/95 | Section I.6 | Goals of monitoring activities  Components of the monitoring plan |
|  | Hydrocarbons | Disp. 123/06 | Section 3.4 | Goals of the monitoring activities  Guidance for selecting indicators  General guidance on monitoring processes |
|  | Roads | Res. 1604/2007 | Annex XI | Components of the monitoring plan  General guidance on monitoring processes |
| Brazil | Energy | Ord. 421/2011 | Annex II | Components of the monitoring plan |
| Chile | General EIA | Decree 40 | Art. 105 | Components of the monitoring plan |
| Colombia* | Hydrocarbons | Res. 1544 | Section 8 | List of environmental elements to monitor |
|  | Energy | Res. 1280 | Section 8 | Goals of the monitoring activities  Components of the monitoring plan  List of environmental elements to monitor |
|  | Energy | Res. 1288 | Section 8 | Goals of the monitoring activities  Components of the monitoring plan |
|  | Energy | Res. 1284 | Section 8 | Goals of the monitoring activities  List of environmental elements to monitor |
|  | General EIA | Decree 2820 | Art.39 | Goals of the monitoring activities  Components of the monitoring plan  Responsible: environmental authority |
|  | General EIA | Res. 1503 | Section 2.6 | Goals of the monitoring activities  Components of the monitoring plan |
| Mexico | Mining | NOM-116-SEMARNAT-2005 | Section 5 | Components of the monitoring plan  Responsible: environmental authority |
|  | Waste | Waste reg. | Art.150 | General guidance on monitoring processes |
|  | Energy | NOM-150-SEMARNAT-2006 | Section 5.4 | Components of the monitoring plan |
|  | Hydrocarbons | NOM-149-SEMARNAT-2006 | Section 6 | Components of the monitoring plan |
|  | Mining | NOM-120-SEMARNAT-2011 | Sections 4.1.19 and 6 | Components of the monitoring plan  Performance standards to evaluate mitigation measures |
| Peru | Waste | SD 057-2004-PCM | Art.92.4 | Responsible: land owner |
|  | Mining | SD 033-2005-EM | Art.24 | Components of the monitoring plan |
|  | Mining | SD 059-2005-EM | Art.43 | Components of the monitoring plan |
| **TIMING PROVISIONS** | | | | |
| **Country** | **Kind of policy** | **Document** | **Section** | **Kind of guidance** |
| Argentina | Protected Areas | Res. 16/94 | Art.18.6.b | Time of data collection |
|  | Hydrocarbons | Disp. 123/06 | Section 2.6 | Time of delivering monitoring reports |
| Mexico | Habitat specific | NOM-022-SEMARNAT-2003 | Section 4.41 | Duration: 3-5 years after measure implementation |
|  | Mining | NOM-116-SEMARNAT-2005 | Section 5 | Time of data collection |
|  | Energy | NOM-150-SEMARNAT-2006 | Section 5.4 | Time of data collection |
|  | Hydrocarbons | NOM-149-SEMARNAT-2006 | Section 6 | Time of data collection |
| Peru | Waste | SD 057-2004-PCM | Art.92.4 | Duration: 5-10 years after project closure |
|  | Mining | SD 059-2005-EM | Art.45 | Duration: ≥5 years after project finishing or closure |

Dec.: decree, Res.: resolution, Disp.: disposition, NI: Normative Instruction, NOM: Official Mexican Rule, SD: Supreme Decree

* Colombia: Resolutions 1543 (hydrocarbons), 1288 (energy), and 1284 (energy), provide the same guidance as Resolution 1544 (in the table), in the same section.

Resolution 1287 (energy) provides the same guidance as Resolution 1280 (in the table), in the same section.

Resolution 1271 (railways) provides the same guidance as Resolution 1288 (in the table), in the same section.

Resolutions 1276 (airports), 1269, 1283, 1289, 1559 (roads), 1253, 1275 (hydrocarbons), 1272 (marine dredging), 1281 (marine ports), 1273 (estuarine dredging), and 1290 (river docks), provide the same guidance as Resolution 1284 (in the table), in the same section.
