# Supplementary material for: Policy Development for Environmental Licensing and Biodiversity Offsets in Latin America
Source: PLoS One. 2014 Sep 5;9(9):e107144. doi: 10.1371/journal.pone.0107144 (PMC4156437; doi:10.1371/journal.pone.0107144)
Supplement: Appendix S1 — List of sources. (DOCX) [file pone.0107144.s006.docx]

Appendix S1. List of sources.

1. **Websites of the Ministries of the Environment and other Government agencies**

| **Country** | **Agency** | **URL** |
| --- | --- | --- |
| Argentina | Secretaría de Ambiente y Desarrollo Sustentable de la Nación | http://www.ambiente.gov.ar/ |
|  | InfoLEG: Información Legislativa y Documental (Ministerio de Economía y Finanzas Públicas) | http://www.infoleg.gob.ar/ |
| Brazil | Ministério do Meio Ambiente | http://www.mma.gov.br/ |
|  | Instituto Brasileiro do Meio Ambiente e dos Recursos Naturais Renováveis (IBAMA) | http://www.ibama.gov.br/ |
| Chile | Servicio de Evaluación Ambiental | http://www.sea.gob.cl/ |
|  | Ley Chile: Biblioteca del Congreso Nacional de Chile | http://www.leychile.cl/Consulta/homebasico |
| Colombia | Ministerio de Ambiente y Desarrollo Sostenible | http://www.minambiente.gov.co/ |
|  | Autoridad Nacional de Licencias Ambientales | http://www.anla.gov.co |
| Mexico | Secretaría de Medio Ambiente y Recursos Naturales | http://www.semarnat.gob.mx/ |
| Peru | Ministerio del Ambiente | http://www.minam.gob.pe |
| Venezuela | Ministerio del Poder Popular para el Ambiente | http://www.minamb.gob.ve/ |

1. **Published articles and reports**

General

Darbi M, Ohlenburg H, Herberg A, Wende W, Skambracks D, & Herbert M. 2009. International Approaches to Compensation for Impacts on Biological Diversity. Final Report. Dresden, Berlin. URL: <http://www.forest-trends.org/publication_details.php?publicationID=522>

Donelly, A., Dalal-Clayton, B., Hughes, R. 1998. A directory of Impact Assessment Guidelines (Second). Nottingham: Russell Press. URL: http://pubs.iied.org/pdfs/7785IIED.pdf?

Environmental Law Alliance Worldwide (eLAW), 2013. EIA Legal Frameworks. URL: <http://eialaws.elaw.org/>

Espinoza G, Alzina V. 2001. Review of Environmental Impact Assessment in Selected Countries of Latin America and the Caribbean. Methodology, Results, and Trends. (G. Espinoza & V. Alzina, Eds.) (p. 90). Santiago de Chile: Inter-American Development Bank (IDB) - Center for Development Studies. URL: http://www.bvsde.paho.org/bvsacd/cd08/review.pdf

Madsen, B., Carroll, N., & Moore Brands, K. 2010. State of biodiversity markets report: offset and compensation programs worldwide. URL: [www.ecosystemmarketplace.com](http://www.ecosystemmarketplace.com)

Madsen, B., Carroll, N., Kandy, D., & Bennett, G. (2011). 2011 Update: State of Biodiversity Markets (p. 39). Washington, D.C. URL: [www.ecosystemmarketplace.com](http://www.ecosystemmarketplace.com)

Argentina

Gabutti E, 2005. Legislación Ambiental de la República Argentina. Centro de Gestión Ambiental, Universidad Nacional de San Luis. URL: <http://www.fices.unsl.edu.ar/cga/legistlacionambra.pdf>

Brazil

Escorcio Bezerra, L. G. 2007. Biodiversity Offsets in National (Brazil) and Regional (EU) Mandatory Arrangements: Towards an International Regime? University College London, Department of Laws. MSc Dissertation.

Glasson, J., & Salvador, N. 2000. EIA in Brazil: a procedures–practice gap. A comparative study with reference to the European Union, and especially the UK. Environmental Impact Assessment Review, 20, 191–225.

Venezuela

PlaniGestión, 2013. Listado de Legislación Ambiental Venezolana. URL: [http://planigestion.com/Documentos/Listado Legislacion Ambiental.pdf](http://planigestion.com/Documentos/Listado%20Legislacion%20Ambiental.pdf)

Embassy of the Bolivarian Republic of Venezuela, n.d. Legislación ambiental venezolana y normas afines. URL: <http://venezuela-us.org/es/wp-content/uploads/2009/09/legislacion-ambiental-venezolana.pdf>

1. **Persons interviewed**

Argentina: Gustavo Iglesias (The Nature Conservancy)

Brazil: Gustavo Pinheiro (The Nature Conservancy), Karen Oliveira (The Nature Conservancy)

Colombia: José Yunis (The Nature Conservancy), Felipe Osorio (The Nature Conservancy)

Chile: Francisco Solís (The Nature Conservancy)

México: Jacquelin Gutiérrez (Newbridge), Mauricio Trejo (Comisión Nacional de Áreas Naturales Protegidas)

Perú: Luis Alberto González (The Nature Conservancy), Óscar Castillo (Wildlife Conservation Society)

Venezuela: Lila Gil (The Nature Conservancy)
