# Supplementary material for: Policy Development for Environmental Licensing and Biodiversity Offsets in Latin America
Source: PLoS One. 2014 Sep 5;9(9):e107144. doi: 10.1371/journal.pone.0107144 (PMC4156437; doi:10.1371/journal.pone.0107144)
Supplement: Appendix S2 — Questions used to assess country level environmental impacts assessment process. (DOCX) [file pone.0107144.s007.docx]

Appendix S2. Questions used to assess country level environmental impacts assessment process.

| **#** | **Question wording** | **Will be answered “yes” if…** |
| --- | --- | --- |
| 1 | Is there a national-level EIA law or regulation? | There is a law or regulation that, at least, sets the bases for EIA processes nationwide |
| 1b | Are there also sector or habitat-specific EIA laws or regulations? | There is a policy that makes provisions for EIA related to specific projects or areas |
| 2 | Are all habitats covered by the EIA law? | There are no restrictions to the habitats covered by EIA laws/regulations |
| 3 | Are ecosystem services addressed at any point? | Reviewed policies at least name the concept |
| 4 | Does EIA explicitly have a no-net-loss or net-gain goal? | Either no-net-loss or net-gain are explicitly established as the objectives of EIA processes |
| 5 | Does the law establish the elements that should be included in the Environmental Impact Study (EIS)? | Any of the reviewed policies lists a series of points that shall be included in EISs |
| 6 | Are impacts assessed at landscape scale? | There is a specific requirement to evaluate impacts from a broad perspective (sector or landscape scale) |
| 6b | Is this level of assessment required for all projects/habitats? | The former requirement applies to all EIAs |
| 7 | Are indirect impacts addressed (at least in some cases)? | There is a specific requirement to evaluate indirect impacts |
| 7b | Are indirect impacts required to be addressed in all EIAs? | The former requirement applies to all EIAs |
| 8 | Are cumulative impacts addressed (at least in some cases)? | There is a specific requirement to evaluate cumulative impacts |
| 8b | Are cumulative impacts required to be addressed in all EIAs? | The former requirement applies to all EIAs |
| 9 | Is there any guidance on how to estimate the significance of impacts? | There is information about how impacts shall be evaluated, e.g. a list of criteria, recommended methods, "best practices"… |
| 9b | Does this guidance affect all EIAs? | The former guidance applies to all EIAs |
| 9c | Does it state (at least in some cases) how different factors should be weighed? | The former guidance specifically states or describes how impact significance shall be calculated, including how to measure criteria related to it |
| 10 | Is the mitigation hierarchy named in the EIA law/regulation? | The three/four steps (avoidance, minimization/restoration, offset) of the mitigation hierarchy are named using proper terms |
| 11 | Are offsets explicitly required at least for some projects/impacts? | There is an explicit requirement to apply offsets for certain projects or impacts, or in certain areas |
| 11b | Are offsets explicitly required for all infrastructure projects subject to EIA, in all cases? | The former requirement applies to all EIAs |
| 12 | Are there any criteria on how to implement avoidance measures or thresholds? | There is a policy that provides some guidance on impact avoidance, including the prohibition of certain activities or of affecting certain areas |
| 12b | Do these criteria go beyond prohibiting impacts on protected areas (at least in some cases)? | The former guidance includes provisions for non-protected areas |
| 12c | Are such criteria set in an EIA general law or regulation? | Some avoidance guidance can be found in national EIA policies that have general scope (not just project or habitat-specific regulations) |
| 12d | Is there any guidance on spatial attributes of avoidance measures (e.g. size of project features)? | Recommendations on the size or location of certain activities or project elements are set to avoid impacts |
| 12e | For all projects subject to EIA, in all areas? | The former provisions apply to all EIAs and are not limited to specific areas |
| 12f | Does this guidance set a list of recommended actions or recommended performance? | Policies provide a list of activities to avoid or to carry out in a specific way to avoid impacts |
| 12g | For all projects subject to EIA, in all areas? | The former provisions apply to all EIAs and are not limited to specific areas |
| 13 | Are there any criteria on how to implement minimization or restoration measures? | There is a policy that provides guidance on impact minimization or restoration (beyond enabling their use for mitigation) |
| 13b | Are there criteria for all projects subject to EIA? | There is guidance on minimization-restoration for all EIAs (does not have to be necessarily set in one single document) |
| 13c | Does this guidance set a list of recommended actions or recommended performance? | Policies provide a list of recommended minimization-restoration measures |
| 13d | For all projects subject to EIA, in all areas? | The former provisions apply to all EIAs and are not limited to specific areas |
| 13e | Does the guidance establish measurable requirements? | Policies establish a measurable way of determining the success of minimization-restoration measures |
| 13f | For all projects subject to EIA, in all areas? | The former provisions apply to all EIAs and are not limited to specific areas |
| 14 | Are there any criteria on how to implement offsets? | Policies provide some guidance on offsets |
| 14b | Are there criteria for all projects subject to EIA? | There is some guidance on offsets for all EIAs (does not have to be necessarily set in one single document) |
| 14c | Is there any guidance on the location of offsets? | Policies set specific provisions on where offsets shall be located |
| 14d | For all projects subject to EIA, in all areas? | The former provisions apply to all EIAs and are not limited to specific areas |
| 14e | Does this guidance set a list of recommended actions? | Policies provide a list of recommended offsets |
| 14f | For all projects subject to EIA, in all areas? | The former provisions apply to all EIAs and are not limited to specific areas |
| 14g | Does the guidance establish measurable success requirements? | Policies establish a measurable way of determining the success of offsets |
| 14h | For all projects subject to EIA, in all areas? | The former provisions apply to all EIAs and are not limited to specific areas |
| 15 | Is there any specific regulation on offsets? | There is at least one national policy that deals specifically with environmental offsets |
| 15b | Is that a general regulation? | The former policy has a broad scope, i.e. it applies to a wide range of projects and/or habitats |
| 16 | Does the law require monitoring mitigation measures? | There is an explicit requirement to perform a follow-up of the mitigation measures after their implementation |
| 16b | Does this requirement apply to all EIAs? | The former provisions apply to all EIAs and are not limited to specific areas |
| 17 | Is there an explicit requirement for monitoring impacts (at least in some cases)? | There is an explicit requirement to perform a follow-up of the predicted impacts after the project has been implemented |
| 17b | For all projects subject to EIA, in all areas? | The former provisions apply to all EIAs and are not limited to specific areas |
| 18 | Is there any guidance on how to perform post-project monitoring? | There are any provisions on how post-project monitoring shall be performed, e.g. factors/criteria to take into account, information the monitoring plan shall include |
| 18b | Does this guidance affect all EIAs? | The former guidance applies to all EIAs and is not limited to specific areas |
| 18c | Are there any provisions regarding elements to address during monitoring? | A list of environmental assets, project activities or impacts to be addressed during monitoring is included |
| 18d | For all projects subject to EIA, in all areas? | The former guidance applies to all EIAs and is not limited to specific areas |
| 18e | Does this guidance establish minimum duration of monitoring activities? | The minimum period during which monitoring activities shall be performed is established |
| 18f | For all projects subject to EIA, in all areas? | The former guidance applies to all EIAs and is not limited to specific areas |
| 18g | Does the policy establish the standards towards which monitoring should be performed? | It is stated in which circumstances a certain monitored element can be considered to have met its intended goals or performance |
| 18h | For all projects subject to EIA, in all areas? | The former guidance applies to all EIAs and is not limited to specific areas |
| 18i | Is monitoring explicitly required also after the closure of the project? | There is an explicit requirement to perform monitoring tasks after the project has been dismantled or the activity has finished |

*Sub-questions (e.g. 1b, 9c, 12d) were only asked if the first question was answered in the affirmative. If the main question is answered “no”, all the following sub-questions were answered “no” as well. In the assessments directed at sectorial activities it was not appropriate to use all the questions. The review of sectorial laws and regulations has not included the following questions and sub-questions: 1, 1b, 6b, 7b, 8b, 9b, 11b, 12c, 12e, 12g, 13b, 13d, 13f, 14b, 14d, 14f, 14h, 15b, 16b, 17b, 18b, 18d, 18f, 18h. An additional question was added in assessment of sectorial activities (numbered as 2b) that was intended to evaluate whether all the main activities of the sector are covered by the reviewed policies. Those activities have been defined for each sector as follows:

- Mining: prospection / exploration, perforation, open mining
- Hydrocarbons: prospection / exploration, drilling / exploitation, transportation (pipes), processing (refineries)
- Energy (electricity): hydroelectric, thermoelectric, nuclear, wind, solar, electric lines
- Transport: roads, railways, airports, ports
- Waste: landfills, waste treatment facilities

Question 2b will be answered “yes” for sectorial assessments if either a general law exists (e.g. Mining Code) which covers all the main activities related to the sector, or there are several more specific policies that together cover all those activities.

Questions have been grouped depending on which aspect of mitigation they cover, as follows:

| **Topic** | **Questions included** (for sectorial assessments the questions pointed out above are excluded) |
| --- | --- |
| Impact assessment | 1, 1b, 2, 2b, 3, 4, 5, 6, 6b, 7, 7b, 8, 8b, 9, 9b, 9c, 10 |
| Avoidance | 12, 12b, 12c, 12d, 12e, 12f, 12g |
| Minimization-restoration | 13, 13b, 13c, 13d, 13e, 13f |
| Offsets | 11, 11b, 14, 14b, 14c, 14d, 14e, 14f, 14g, 14h, 15, 15b |
| Monitoring | 16, 16b, 17, 17b, 18, 18b, 18c, 18d, 18e, 18f, 18g, 18h, 18i |

It is noticeable that not all the categories have the same number of questions. There are more questions related to impact assessment and to monitoring because these are broader topics than the other three. Also, there is a high number of questions related to offsets because this is the main focus of the paper.
